# What we have learned from a patient with partial tracheal rupture caused by penetrating neck injuries: a case report

**DOI:** 10.1186/s12871-022-01886-0

**Published:** 2022-11-01

**Authors:** Jun Tian, Xing Tao, Xiang Quan, Sanmei Zhang

**Affiliations:** 1 Department of Otolaryngology, Head & Neck Surgery, Beijing Friendship Hospital, Capital Medical University, No. 95 Yongan Road, Xicheng District, Beijing City, 100050 China; 2 Department of Anesthesiology, Beijing Friendship Hospital, Capital Medical University, Beijing City, China; 3 Department of Anesthesiology, Peking Union Medical College Hospital,Chinese Academy of Medical Science and Peking Union Medical College, Beijing City, China; 4 Department of Medical Insurance, Beijing Friendship Hospital, Capital Medical University, Beijing City, China

**Keywords:** Penetrating neck injuries, Airway management, Rescue tracheotomy, Difficult airway, Case report

## Abstract

**Background:**

Airway management of patients with direct airway trauma caused by penetrating neck injuries is always challenging. When a failed airway occurs and surgery access is difficult, it is crucial to find the optimal approach to save the life. We propose the concept “Cannot intubate, Cannot oxygenate, Difficult surgery access” to describe this emergency scenario.

**Case presentation:**

We report a case of a 24-year-old woman who presented with partial tracheal rupture and pneumothorax caused by a knife stab injury to the neck. A "double setup" strategy, simultaneous preparation for orotracheal intubation and tracheotomy, was carried out before rapid sequence induction. A tracheotomy under local anesthesia or an awake intubation was not preferred in consideration that the patient had a high risk of being uncooperative owing to existing mental disease and potential smothering sensation during operation. During rapid sequence intubation, distal part of the tube penetrates the tear and creates a false lumen outside the trachea then a failed airway subsequently occurred. Rescue tracheotomy was successfully performed by an otolaryngology surgeon, with the help of limited ventilation using sequential bag–mask and laryngeal mask airway ventilation provided by an anesthesiologist, without severe sequelae.

**Conclusions:**

The endotracheal tube have a risk of penetrating the tear outside the trachea in patient with partial tracheal rupture during orotracheal intubation, and once it occurs, proceeding directly to an emergency invasive airway access with optimizing oxygenation throughout procedure might increase the chance of success in rescuing the airway.

## Background

Penetrating neck injuries (PNIs) are life-threatening due to the severe aerodigestive and neurovascular trauma. Mortality from penetrating laryngotracheal trauma is reported to be as high as 20% [1]. Successful emergency airway management in patients with direct airway trauma owing to PNIs is challenging. In cases of hypolaryngeal injury, once a failed airway has occurred and emergency invasive airway access has been indicated—since the relatively easier emergency scalpel cricothyrotomy is not effective—the expeditious surgical tracheotomy as the last resort is also always difficult.

It is known that the combination of an anticipated difficult intubation and the inability to maintain SpO_2_ at adequate levels is a surrogate for the “cannot intubate, cannot ventilate” failed airway. As for a distorted airway, “difficult surgery access” should be added to this professional term. There are no guidelines or expert advice on what the best approach and technique for airway protection is to avoid the serious airway-related morbidity and mortality related to this critical scenario. Herein, we report a case of successful failed airway treatment after rapid sequence intubation (RSI) of a false passage to the trachea in a patient with partial tracheal rupture caused by stab injuries to the neck. Combined anesthetic and surgical expertise is required in this situation. Although a number of guidelines and algorithms exist for difficult airway management of direct airway trauma, a distorted airway, or PNIs, all the relevant techniques are likely difficult and may fail. The pearls and pitfalls concluded from the perspective of both surgeons and anesthesiologist in the case may elucidate the management of similar complicated situations.

## Case presentation

**Fig. 1:**
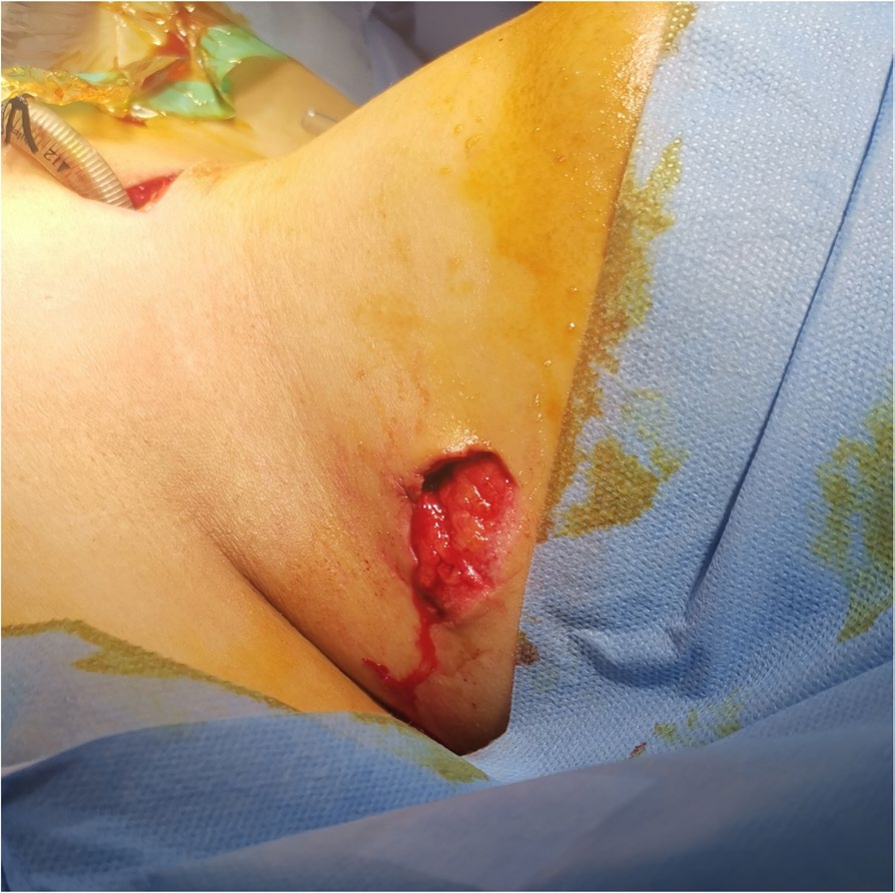
Clinical examination shows a 1 cm-long skin wound in the upper left neck

**Fig. 2:**
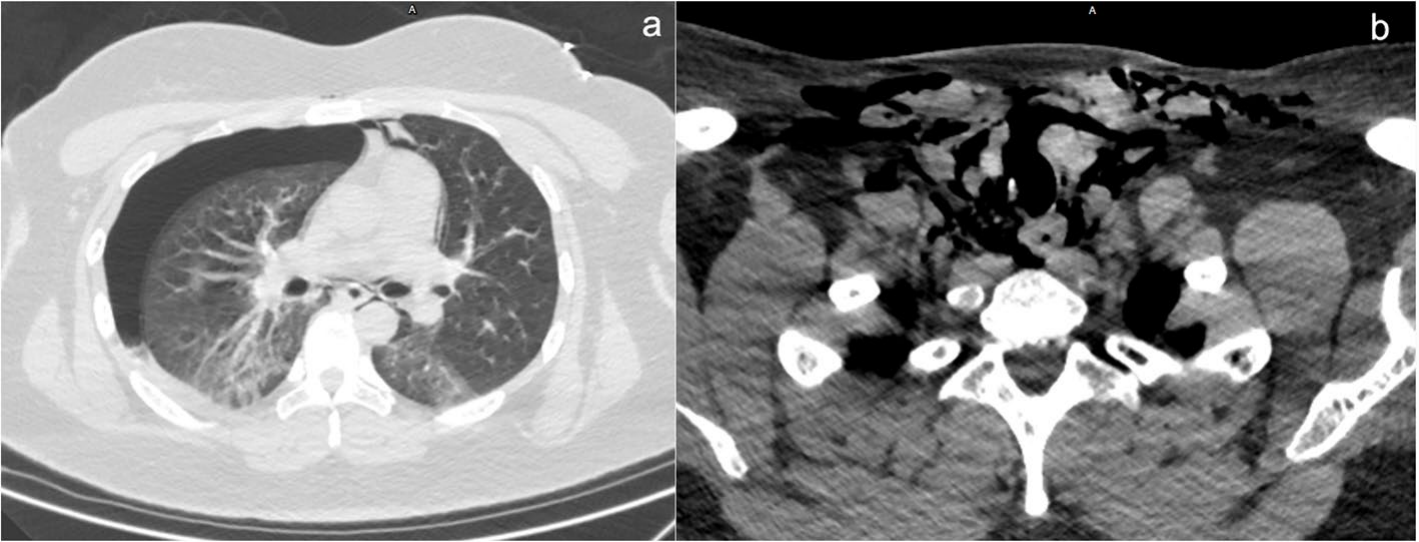
Chest and cervical computed tomography results. **a** Right pneumothorax; **b** Severe subcutaneous pneumatosis and partial rupture of the trachea at the level of thyroid isthmus

A 24-year-old woman was admitted to the emergency department of Beijing Friendship Hospital of Capital Medical University 4 h after receiving a knife stab injury to the neck due to urban violence. The patient weighed 98 kg and had a history of bipolar disorder. Upon arrival at the hospital, her level of consciousness was normal. She presented with a hoarse voice, intermittent hemoptysis, and slight mixed dyspnea during quiet respiration without hypoxemia. Clinical examination showed a 1 cm-long skin wound in the upper left neck with an intermittent hemorrhage (Fig. 1), decreased breath sounds on the right side, and palpable subcutaneous emphysema. Chest and cervical computed tomography (CT) showed right pneumothorax (Fig. 2a), severe subcutaneous pneumatosis, and partial rupture of the trachea at the level of the thyroid isthmus (Fig. 2b). Active bleeding was controlled with the appropriate amount and direction of direct pressure using gauze. A difficult airway was anticipated and the patient was expeditiously transferred to the operating room to avoid airway compromise and worsening hemorrhage rapidly.

First, aimed at providing sufficient respiratory function reserve for oncoming risks during airway management, closed thoracic drainage under local anesthesia was performed. Rapid sequence induction and intubation as opposed to awake intubation was chosen based on the following considerations: 1. The patient had a high risk of being uncooperative owing to existing mental disease. 2. Awake intubation was also a high-risk approach—rapid airway deterioration might have occurred under sedation and topical anesthesia, and during flexible laryngoscopy, due to an excessive coughing reaction and increased airway bleeding. Excessive blood or secretions in the airway might have limited the effectiveness of the topical anesthetics and adequately deep sedation without topical anesthesia would not have been achieved. The presence of copious amounts of tracheal blood would have likely rendered flexible fiberoptic laryngoscopy difficult or impossible. A longer-than-normal approach might have placed the patient in danger since she initially appeared stable but could have decompensated rapidly. 3. A fiberoptic bronchoscope with a large suction port was not available.

Similar to awake intubation, a tracheotomy under local anesthesia was anticipated challenging and difficult for several reasons. First of all, the patient had a high risk of being uncooperative owing to existing mental disease. Secondly, we have to consider other risk factors under whatever anesthesia, such as unknown vascular injury, distorted anatomy, hemostasis, airway protection from bleeding and external pressure during operation, patient position and longer than normal duration of operation. Above all, we don’t think that the risk of the failure of airway during tracheotomy under local anesthesia initially is lower than that after failure of transoral intubation. And if intubation succeed, it avoid placing the patient in danger due to airway compromise. So, we did not prefer tracheotomy under local anesthesia in this patient.

**Fig. 3:**
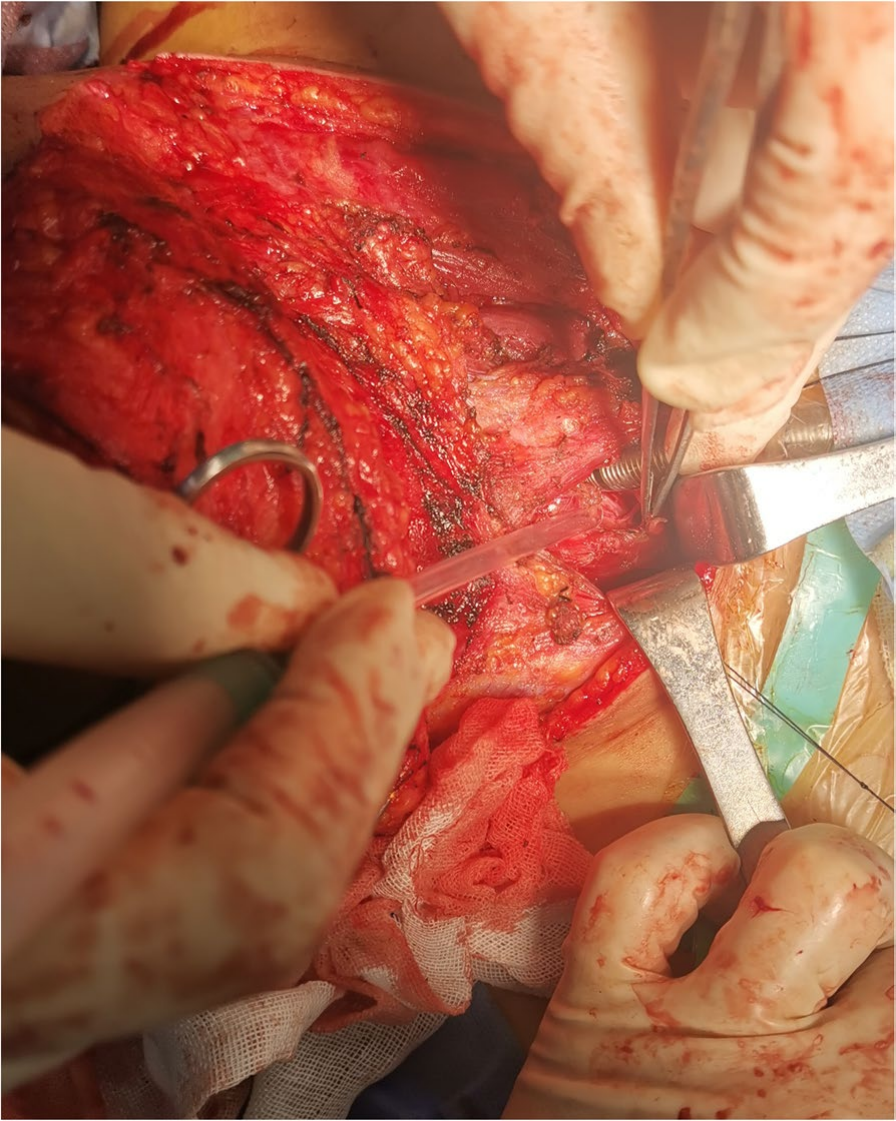
Operative exploration results. The thyroid isthmus and right anterior tracheal wall are partially ruptured. The right recurrent laryngeal nerve is transected

Before beginning the intubation sequence, communication between the anesthesiologist and surgeon focused on the risk that a preexisting anatomic defect in the patient’s trachea made the endotracheal tube more apt to penetrate the tear and be introduced into a false lumen. Consequently, a double setup, where both an alternative airway device and surgical airway were identified and readied, was used. After preoxygenation for five minutes as usual and rapid sequence induction using midazolam, sulfentanyl, etomidate and rocuronium bromide, the patient’s SpO2 level decreased to 70% in 40 s, Transoral intubation with video laryngoscopy but with no cricoid pressure was administered quickly; however, after successfully passing through the glottis, resistance was encountered. The patient’s SpO_2_ level continued decreasing to 40% after replenishing oxygen. The tracheal tube was thought to have been introduced into a false lumen passage and the patient was rapidly extubated. Simultaneously, a surgical procedure was initiated. During surgery, an Laryngeal Mask Airway (LMA) was placed with facilitated bag-mask ventilation, which maintained the SpO_2_ level at approximately 80% while the surgeon cut the skin, provided hemostasis, and identified distorted anatomical landmarks. When the pretracheal fascia was nearly exposed, the SpO_2_ level suddenly decreased to lower than 10%; the broken end of trachea was roughly identified, and the tube was introduced into the distal trachea with no further reduction in SpO2. After an adequate SpO_2_ level was maintained, a penetrating injury from the upper left neck to the right apex of the chest was found. The thyroid isthmus and right anterior tracheal wall were ruptured (Fig. 3) and the right recurrent laryngeal nerve transected. Bleeding originated mainly from the thyroid vessels.

**Fig. 4:**
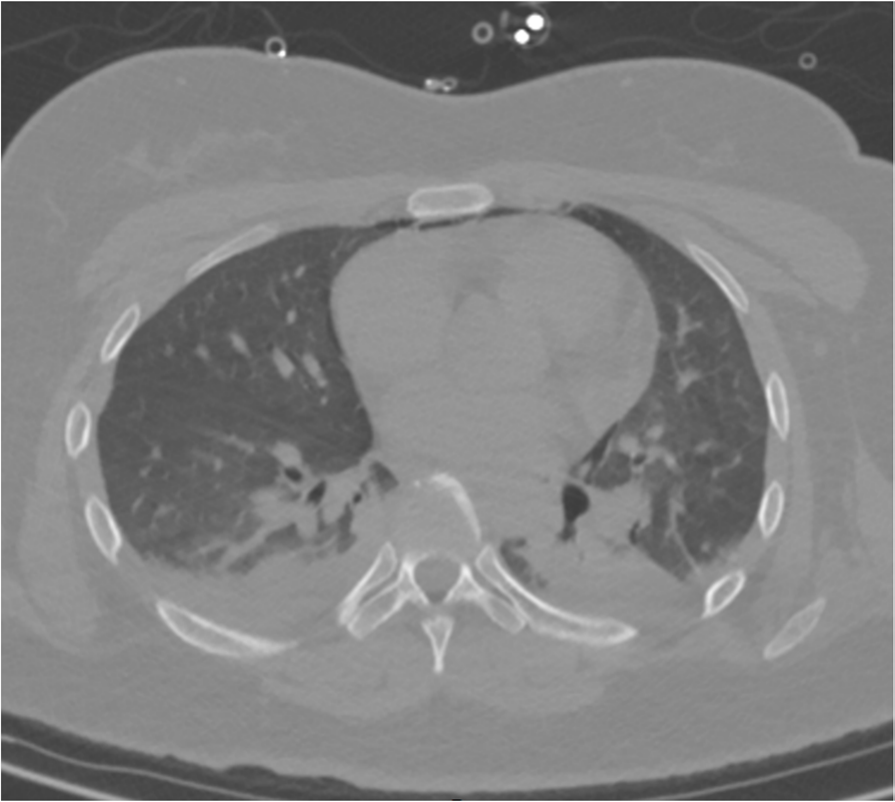
Postoperative chest computed tomography showing aspiration pneumonia

**Fig. 5:**
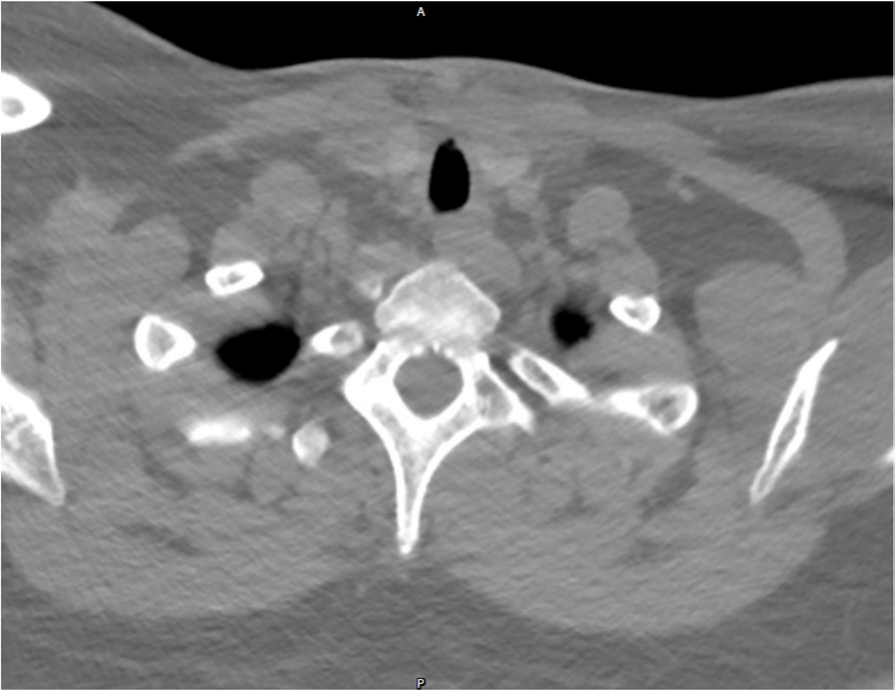
Cervical computed tomography results. After two weeks of hospitalization, showing tracheal intubation was successfully removed and tracheostomy healed without the need for further reconstruction surgery

After the surgery, the patient recovered from anesthesia without impairment of brain function. Postoperative chest CT showed aspiration pneumonia (Fig. 4). After 2 weeks of hospitalization, the tracheal tube was successfully removed and the tracheostomy was repaired by pulling the skin using medical tape, without the need for further reconstruction surgery (Fig. 5).

## Discussion and conclusions

It is reasonable to be prepared to provide a surgical airway immediately if attempts at oral intubation fail. However, it should be noted that timely establishment of a surgical airway in some patients with PNIs is challenging. It is difficult to determine the optimal systematic approach for successful airway management in such an emergency scenario.

Despite the number of different practice guidelines available to assist practitioners with the management of an anticipated [2] or unanticipated difficult airway [3],there are no optimal algorithms to follow which adequately deal with a complicated traumatic airway. And there are few details about the options for difficult airway management of uncooperative patient in some guidelines. All techniques used to manage these kind of scenarios are likely to be difficult and may fail. Mercer et al. [4] have suggested management options for Non-iatrogenic trauma to the airway. And the major feature is to divide the patients with trachea and bronchi trauma into two groups: cooperative patient and uncooperative patient. However, it should be proved in clinical practice with further research.

In an emergency difficult airway management, validation of a simple algorithm to follow in daily practice is the key to success in emergencies. Vortex, a common cognitive aid that facilitates decision making and communication in real time during airway emergencies [5], suggests that if a best attempt at either face mask ventilation, orotracheal intubation, or placement of a supraglottic device is unsuccessful (< 3 attempts), the other two candidates should be attempted; if unsuccessful, one should proceed to emergency surgical access. However, each failed attempt may decrease the available time for optimal manipulation and increase the patient’s risk of death. Nevertheless, in such a situation, it is the patient’s critical condition that forces the operator to act immediately to forestall deterioration to respiratory arrest or complete airway obstruction. Every action should be evaluated quickly to determine whether the benefits outweigh the risks. The present case exhibits successful airway management in a patient with a PNI based on collaboration of a temporary airway management team. Although there is a lot of experience to be shared, it highlight the importance of limiting the number of attempts at tracheal intubation to allows more time for performing an invasive airway and supplemental oxygen administration throughout difficult airway management in the face of difficult invasive airwa. It was our simple philosophy followed in this special circumstance, which was also emphasized in 2022 American Society of Anesthesiologists Practice Guidelines for management of the difficult airway [6].

It is difficult to provide definitive conclusions on how to best manage various airway-penetrating injuries, because the diverse nature of these injuries precludes emulating a single method of choice and the sample size of most relevant studies is small [7]. Upon re-examination of this case, despite our best explanations as below, some questions remained. Nonetheless, the presentation and review of each case is important in order for lessons to be learned and future management strategies to be improved.

1. Is RSI or the “awake look” approach more reasonable in a patient with non-exposure partial rupture of the trachea? When the airway is threatened but anatomic structures and relationships are preserved as nearly normal, several observational studies suggest that RSI is effective in patients with PNIs [4]. Some clinicians suggest that an “awake” approach with maintenance of spontaneous ventilation and intubation under direct vision in patients with traumatized airways may be best, especially if the airway assessment suggests that intubation after induction of general anesthesia may fail and/or that the “rescue technique” may be difficult or impossible [8]. However, this technique is usually not possible in combative patients or when immediate airway access is required. Some clinicians suggest the “awake look” approach is prudent considering that it may introduce airway collapse [9]. The concern in the present case was that the patient might have become agitated and uncooperative because of a feeling of suffocation when the first attempt at intervention failed. When the neck trauma is such that it distorts anatomic landmarks and is likely to make effective ventilation or successful placement of a tracheal tube unlikely, a surgical airway is generally recommended. Of note, a primary surgery is still risky and challenging and its success relies on the support from the anesthesiologist.

Nevertheless, the best method to achieve definitive airway control in the setting of PNIs will vary according to the patient’s injuries, attributes, and likelihood of deterioration; the skills and confidence of the airway manager; the degree of anatomical damage based on the judgment of the operator; and the available resources. No matter how high the probability of success using these approaches may be, the emergency rescue tracheotomy as a double setup should be readied.

2. At times, subcutaneous emphysema represents a contraindication to BMV because it may force air into injured tissue planes, distort airway anatomy, and compromise subsequent efforts to ventilate or intubate. For the same reason, supraglottic devices such as the LMA, laryngeal tube, or pharyngeal tube used as rescue techniques may be not effective. However, as shown in the case described in this article, both BMV and LMA ventilation to reoxygenate following a failed attempt at transoral intubation may play an import role in maintaining an acceptable SaO2 as the surgery proceeds, even though its effectiveness is not assured. Gentle, controlled ventilation may be fundamental in ensuring the benefit outweighs the risks.
3. When the “final rescue technique” such as tracheotomy is anticipated to be very difficult, the time redundancy of the rescue surgery is the most important factor for final success. Although the guidelines for difficult airway management suggests three attempts or fewer before transitioning to an alternative approach, repeated procedural attempts should be limited to minimize delays and avoid task fixation [10]. It may be optimal to avoid trying again and save more time for a longer-than-normal tracheotomy considering that, if the clinician loses the contest emerging between the clinician’s efficiency placing the tracheal tube and the patient’s descent along their oxyhemoglobin desaturation curve, the cost of failure is brain anoxia or the death of the patient.
4. Fiberoptic guided oral intubation may be helpful because it enables the clinician to identify the injuries and facilitates the introduction of an endotracheal tube distal to the injury to avoid the creation of a false lumen [11], However, the operator must be prepared for failure because of impeded visibility resulting from severe bleeding, even in experienced hands. In this case, a fiberoptic bronchoscope was available and we intended to place tracheal tube at introitus of vocal cords with videolarygoscopy and advance under direct via fibrescope initially. However, after preoxygenation and repid sequence induction, the SaO2 of the patient dropped under the 70% within 40 s.we did not use fibrescope as expected considering it might take more time. Another concern was that the device available had not a large suction port, which might make it ineffective in the bleeding airway, even in experienced hands. And after the first failed endotracheal intubation, we did not try to use it because the traumatic airway might be further disrupted by the the first intubation attempt. Nevertheless, our choice in this case dose not mean that we deny intubation via fibrescopeit might be an optimal management even in an emergency situation. Schaefer et al. [12] recommend to intubate under direct vision in the management of the traumatic airway, since they observed a range of injuries induced by intubation, which included avulsion of the endolaryngeal mucous membrane, creation of pseudo-lumens or false passages, disruptions of tenuous airways by the endotracheal tube, and respiratory arrest.
5. Confidence of both anesthesiologists and surgeons in the airway management of patients with PNIs is one of the major factors influencing decision making and operation. Naturally, the confidence is based on training in both the technical procedure for performing flexible fiberoptic endoscopy and decision-making aspects concerning the failed airway, with regular reinforcement and practice. The otolaryngology surgeon in the present case reported that his confidence had not collapsed mainly because of the support from the anesthesiologist during tracheotomy. Although rescue BMV and LMA ventilation did not maintain the SaO_2_ above 90% during the neck access surgery, a relatively stable oxygen saturation relieved the time pressure dramatically and provided the surgeon with enough confidence to find the tracheal rupture amidst a massive hemorrhage obscuring their vision.

### Lessons

The case presented in this article suggested that the preoxygenation as usual might not adequate for a patient with traumatic airway. And the use of neuromuscular blockade maybe controversial because muscle tone may be important for airway integrity in airway transectionit and it theoretically result in airway collapse [13]. The optimal dose of neuromuscular blocking agents during rapid sequence induction for tracheal injuries should be further studied.

In conclusion, the airway management of the direct airway injury as a result of neck penetration is challenging. The endotracheal tube have a risk of penetrating the tear outside the trachea in patient with partial tracheal rupture during orotracheal intubation, When difficult invasive airway is anticapted and the worst situation occurs ( Cannot intubate, Cannot oxygenate), proceeding directly to an emergency invasive airway access with optimizing oxygenation throughout procedure might increase the chance of success in rescuing the airway.

## Data Availability

The datasets used and/or analysed during the current study are available from the corresponding author on reasonable request.

## Abbreviations

BMV: Bag–mask ventilation
CT: Computed tomography
LMA: Laryngeal mask airway
PNIs: Penetrating neck injuries
RSI: Rapid sequence intubation
